# Single‐parentage assignments reveal negative‐assortative mating in an endangered salmonid

**DOI:** 10.1002/ece3.8846

**Published:** 2022-04-25

**Authors:** Craig A. Steele, Thomas A. Delomas, Matthew R. Campbell, John H. Powell

**Affiliations:** ^1^ Pacific States Marine Fisheries Commission Eagle Fish Genetics Lab Eagle Idaho USA; ^2^ 115870 Idaho Department of Fish and Game Eagle Idaho USA; ^3^ 115870 Idaho Department of Fish and Game Boise Idaho USA; ^4^ Present address: USDA ARS National Cold Water Marine Aquaculture Center Kingston Rhode Island USA

**Keywords:** mating behavior, parentage assignment, sockeye

## Abstract

Understanding reproductive patterns in endangered species is critical for supporting their recovery efforts. In this study we use a combination of paired‐parent and single‐parent assignments to examine the reproductive patterns in an endangered population of sockeye salmon (*Oncorhynchus nerka*) that uses Redfish Lake in central Idaho as a spawning and nursery lake. Recovery efforts include the release of maturing adults into the lake for volitional spawning. The lake is also inhabited by a population of resident *O*. *nerka* that is genetically indistinguishable, but phenotypically smaller, to the maturing adults released into the lake. The resident population is difficult to sample and the reproductive patterns between the two groups are unknown. We used results of paired‐ and single‐parentage assignments to specifically examine the reproductive patterns of male fish released into the lake under an equal sex ratio and a male‐biased sex ratio. Assignment results of offspring leaving the lake indicated a reproductive shift by males under the two scenarios. Males displayed an assortative mating pattern under an equal sex ratio and spawned almost exclusively with the released females. Under a male‐biased sex ratio most males shifted to a negative‐assortative mating pattern and spawned with smaller females from the resident population. These males were younger and smaller than males that spawned with released females suggesting they were unable to compete with larger males for spawning opportunities with the larger, released females. The results provided insights into the reproductive behavior of this endangered population and has implications for recovery efforts.

## Graphical abstract

Parentage assignments indicated a reproductive shift by male sockeye salmon under two different sex ratios. Males displayed an assortative mating pattern under an equal sex ratio and spawned almost exclusively with similarly sized females but shifted to a negative‐assortative mating pattern under a male‐biased sex ratio and spawned with smaller females in the resident population.

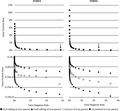

## 1 INTRODUCTION

Understanding the reproductive patterns of endangered species is critical to the preservation of sustainable wild populations and genetic analyses are increasingly used to provide advances in conservation and recovery efforts (Comizzoli & Holt, 2019). The population of sockeye salmon (*Oncorhynchus nerka*) that spawn and rear in Redfish Lake in central Idaho, USA were federally listed as endangered in 1991 under the Endangered Species Act (ESA; 16 USC §1531). Recovery efforts for this evolutionary significant unit are undertaken collaboratively through the multi‐agency Stanley Basin Sockeye Technical Oversight Committee (Kline & Flagg, 2014). As a component of these efforts, the Idaho Department of Fish and Game monitors the reproductive success of adults released above a barrier weir into Redfish Lake for volitional spawning (Kozfkay et al., 2019). Released fish comprise a combination of anadromous adults and captive‐reared (i.e., released) adults from two conservation hatcheries. The lake is also inhabited by a resident population of *O*. *nerka* (Winans et al., 1996) which is smaller in size, but not genetically differentiated, from the released individuals (Cummings et al., 1997; Waples et al., 2011). These resident fish cannot easily be sampled for analyses but spawn at the same time and place as the released population (Brannon et al., 1994) and contribute to the production of migratory smolts (Waples et al., 2011).

Mating patterns between the two groups of spawners (adults released into the lake and smaller residents) are unknown but predictions can be made based on theoretical expectations and tested using parentage analyses. Released fish are expected to exhibit size‐assortative mating in which females preferentially spawn with similarly sized males (Foote & Larkin, 1988). However, the number of released females is expected to be a limiting factor for the reproductive success of males resulting in intrasexual competition among the released males (Quinn, 2011). Increases in deviations from an equal sex ratio are predicted to increase intrasexual aggression within the more abundant sex (Emlen & Oring, 1977; Weir et al., 2011) and observed patterns of male:male competition within *O*. *nerka* are consistent with this expectation (Quinn et al., 1996). Additionally, intrasexual competition among male salmonids favors larger individuals, which exclude smaller males from spawning (Fleming & Gross, 1994; Quinn & Foote, 1994). Based on these insights, we predict that under a male‐biased sex ratio the smallest of the released males, in this study, will be excluded from spawning with females released into the lake and instead seek spawning opportunities with the resident females. Even though these males would represent the lower end of the size distribution for the released adults (~400 mm fork length (FL)), the resident females are substantially smaller (200–237 mm FL; Brannon et al., 1994; Johnson & Provecek, 1993; Pravecek & Johnson, 1997), which would result in a negative‐assortative (also called disassortative) mating pattern in which individuals with dissimilar phenotypes tend to mate with one another.

A common method for evaluating reproductive patterns is the use of genetic parentage analysis in which genotypes of offspring are matched to those of a potential pair of parents. Parentage analysis has provided numerous insights into the ecology, evolution, and behavior of organisms (Flanagan & Jones, 2019; Jones et al., 2010), but an obvious limitation is that it requires both parents to be sampled in order to make an assignment. In study systems where some parents are unsampled researchers must rely on approaches that can reliably assign an offspring to just a single parent. Numerous approaches are available to infer a single‐parent assignment including: exclusion based on Mendelian incompatibilities (Bravington et al., 2016), maximum likelihood using programs such as cervus (Kalinowski et al., 2007) or colony (Jones & Wang, 2010), Bayesian approaches using programs like solomon (Christie et al., 2013), and pedigree‐reconstruction programs such as sequoia (Huisman, 2017) or franz (Riester et al., 2009). However, we find that existing methods are often not conducive for efficient workflow because they are computationally intensive with large datasets, do not allow for determining a priori assignment criteria, and do not provide estimates of error rates associated with the single‐parentage assignment. This is especially true when close relatives of the parent (e.g., full‐siblings and half‐siblings) are also present in the dataset. Therefore, we build upon existing methods and use an approach that performs single‐parentage assignments through a pairwise process of calculating the likelihood ratio for a parent–offspring relationship to that of the pair being unrelated (Anderson & Garza, 2006; Baetscher et al., 2018; Marshall et al., 1998; SanCristobal & Chevalet, 1997; Thompson, 1976) and combine it with estimates of the associated error rates for these assignments using stratified sampling (Delomas & Campbell, 2021). This methodology is implemented in the R package grandma (https://github.com/delomast/gRandma). grandma uses allele frequencies from the observed parental genotypes to simulate candidate parent–offspring pairs that have varying degrees of relatedness in order to explore the expected frequency of error rates when close relatives are present in the dataset. This approach allows one to assess whether the molecular dataset can accurately identify single‐parent assignments and allows the user to select an appropriate assignment threshold to minimize the false positive and false negative rates for their study‐specific analysis.

We use a combination of paired‐parent and single‐parent assignments to test predictions of sex‐specific reproductive patterns and mating behavior in the endangered population of Redfish Lake sockeye salmon. Based on previous observations within this species we predict: (a) under an equal sex ratio the males released into the lake will display an assortative mating pattern and spawn with larger females also released into the lake (b) under a male‐biased sex ratio some released males will display a negative‐assortative mating pattern and spawn with smaller resident females, (c) males with a negative‐assortative mating pattern will be smaller than males that spawned assortatively. Results from this study provide insights into the reproductive patterns of this endangered population and have implications for recovery efforts.

## 2 MATERIALS AND METHODS

### 2.1 Parental sex ratios and offspring sampling

Releases of adult fish into Redfish Lake for volitional spawning occur annually but the number of individuals varies based on the abundance of anadromous returns and availability of captive‐reared fish. We examined reproductive patterns in two consecutive spawn years (SY2013 and SY2014), each with differing sex ratios of adults released into the lake. Captive‐reared fish released during these years were propagated following practices outlined for ESA‐listed stocks (Maynard et al., 2012). Prior to their release, the sex and reproductive maturity of all fish was confirmed using ultrasound imaging, fish were genetically sampled, and their FL measured. Because the population is highly managed and intensively monitored it was also possible to use parentage analysis to determine the year in which a released fish's parents were spawned and thereby calculate its age for this study (Kozfkay et al., 2019). Ages and lengths of the males were used to explore differences between assortative and disassortative individuals.

Adults released in the fall of 2013 had a sex ratio skewed towards males. A combination of male (*n* = 238) and female adults (*n* = 108) were released into the lake resulting in a male:female sex ratio over 2:1. Progeny from these parents hatched the following spring, reared for either 1 or 2 years in the lake and then emigrated as smolts the following spring. Emigrating offspring were sampled at the outlet of Redfish Lake in the spring of 2015 and 2016 and analyzed with paired‐parent and single‐parentage analyses to identify offspring originating from the 2013 spawn year.

Adults released in the subsequent spawn year of 2014 had a nearly equal sex ratio. A combination of male (*n* = 977) and female adults (*n* = 1094) were released for volitional spawning resulting in a male:female sex ratio approaching 1:1. Potential progeny from these parents were sampled as emigrating smolts in 2016 and 2017 and analyzed to identify individuals originating from the 2014 spawn year.

All emigrating offspring were sampled using trap boxes situated at a permanent weir site located 1.4 km downstream of the lake outlet. Sampling of offspring for genetic analysis was conducted annually from early April until smolts stop emigrating in mid‐June (Johnson et al., 2016, 2017, 2019). The number of offspring sampled in 2015, 2016, and 2017 was 1502, 2640, and 760, respectively (Table 1).

**TABLE 1:** Assignment results for sampled juveniles. Juveniles emigrate after either one or two years of rearing in Redfish Lake, Idaho

	Juvenile migration year		
Juv. assignment result			
Parent‐Origin	2015	2016	2017
2‐Parent assignment:			
Ma/Pa‐SY2013	153	13	0
Ma/Pa‐SY2014	0	2110	228
1‐Parent assignment:			
Pa‐SY2013	86	3	0
Ma‐SY2013	3	2	0
Pa‐SY2014	0	13	2
Ma‐SY2014	0	174	30
No assignment	1253	318	498
Failed to genotype	7	7	2
Total	1502	2640	760

Note

Offspring from the 2013 spawn year emigrated in 2015 or 2016 and offspring from the 2014 spawn year emigrated in 2016 or 2017. Parental assignments are indicated either as a paired‐parent assignment (Ma/Pa) or a single‐parent assignment (Ma or Pa) to the spawn year of origin. Offspring that received no parental assignments are likely a combination of progeny from the unsampled resident population or progeny of released adults from spawn years that were not part of this study (i.e., SY2012 and SY2015).

### 2.2 Assessing assortative and negative‐assortative mating patterns

Genetic samples could only be collected from larger adults released into the lake. Therefore, the mating patterns of these adults was inferred based on results of paired‐parent and single‐parent assignments of offspring leaving the lake in the following years. For example, if an offspring received a paired‐parent assignment to two released parents then the parents were determined to have mated assortatively (i.e., both parents were adults released into the lake). If an offspring received a single‐parent assignment then that parent must have mated disassortatively (i.e., an adult released into the lake spawned with an unsampled resident individual). If a parent was detected in both a paired‐parent and a single‐parent assignment then it was determined that the parent participated in both spawning strategies (assortative and disassortative). The proportion of assortatively and disassortatively mating males under the two sex ratios was compared. While the null hypothesis is that the proportion of males in each category will be equal under the two different sex ratios, we expect the proportion of males that mate disassortatively (i.e., spawn with smaller resident females) will be higher when the sex ratio of the larger spawners is male‐biased than when the sex ratio is equal.

### 2.3 Laboratory protocol

Genomic DNA from parents and offspring was extracted from fin clips following the methods for the Nexttec Genomic DNA Isolation Kit from XpressBio (Thurmont, Maryland). We genotyped samples at 382 SNPs using target sequences described by Hasselman et al. (2018). SNPs were genotyped using the protocols of library preparation for next‐generation sequencing using the Genotyping‐In‐Thousands (GT‐Seq) methodology (Campbell et al., 2015). Briefly, library preparation begins with an initial multiplex PCR that is used to ligate sequencing primers to the target sequences that are known to contain single‐nucleotide polymorphisms (SNPs). In a subsequent PCR reaction the sample is “barcoded” by ligating an additional sequence to the target that identifies the sample's tray of origin (i7 barcode) and its position on the tray (i5 barcode). After barcoding, the quantity of DNA was normalized for each sample using a SequalPrep™ Normalization Plate Kit (Applied Biosystems) that binds a standard amount of amplicon product for the normalization of concentrations. All samples per tray were then pooled into a single “plate library” that was quantified by a Qubit fluorometer (Thermo Fisher). Concentrations were normalized again before being pooled. Loci were genotyped by sequencing the target location on an Illumina NextSeq. A custom bioinformatics pipeline was used to assign resulting sequences and the genotypes back to individual samples using the unique combination of i5 and i7 barcodes. Standardized genotypes were stored on a Progeny database server (www.progenygenetics.com) housed by the Eagle Fish Genetics Laboratory.

### 2.4 Parentage analyses

Analyses for paired‐parent assignments were conducted with the program snppit (Anderson, 2012). We allowed up to 10% missing genotype data for a sample before excluding it from consideration in parentage. We used an estimated SNP genotyping per allele error rate of 0.5%. snppit assesses confidence of parentage assignments using several criteria including a false discovery rate (FDR) and a log of odds ratio (LOD). We only accepted assignments with a stringent FDR threshold of <0.05% and an LOD of >19. Offspring that did not receive a paired‐parent assignment were then evaluated with single‐parentage assignments.

Functions to perform single‐parent analyses using the method described here are available in an R package we provide (https://github.com/delomast/gRandma). All functions use calculations that assume loci are in Hardy–Weinberg equilibrium and are unlinked. Population allele frequencies, genotyping error rates, and rates of missing genotypes for each locus were estimated and then treated as known. Population allele frequencies were estimated as the posterior means with Beta (0.5, 0.5) priors and the observed allele frequencies of the potential parents. Rates of missing genotypes were calculated as the posterior means with Beta (0.5, 0.5) priors and the observed frequencies of missing genotypes of both potential parents and potential offspring. The genotyping error rate was also assumed to be 0.5% per allele for all loci.

Potential parent–offspring pairs were evaluated using a two‐step process. The first step eliminates potential parent–offspring pairs with Mendelian incompatibilities (MIs) above a calculated threshold value. The maximum number of allowable MIs was calculated for each parental population such that the probability of excluding a true parent–offspring pair was <10^−4^ when all genotypes were observed (no missing data). Pairs with missing data have a lower probability of exclusion because missing data reduces the number of observed MIs. This threshold was calculated following the method described by Anderson (2012). Briefly, the observation of MIs across loci was represented as a Markov chain. The forward step of the forward–backward algorithm was used to calculate the maximum number of allowable MIs, *m*, given the maximum probability of exclusion for a true parent–offspring pair (<10^−4^) and assuming genotypes at all loci were observed.

The second step calculated a log‐likelihood ratio (LLR) comparing the likelihood of observing the genotypes of a potential parent–offspring pair given a true parent–offspring relationship and the likelihood of observing their genotypes if the pair was unrelated (Anderson & Garza, 2006; Baetscher et al., 2018; Thompson, 1976; Thompson, 2000). The true genotypes of individuals are unknown, and so the likelihood of the observed genotypes was calculated using the assumed genotyping error rates to marginalize the true genotypes (Anderson & Garza, 2006). If the LLR was above or equal to a threshold value (*c*) the assignment was accepted, otherwise the assignment was rejected. Values of *c* were selected by evaluating the estimated false positive and false negative error rates associated with a range of potential values (0–20) for *c* in each parental dataset.

### 2.5 Per‐comparison error rate estimation

In order to determine the level of confidence placed in the single‐parent assignments, it is important to estimate two types of error: the false positive rate (i.e., incorrectly assigning an offspring to a non‐parent) and the false negative rate (i.e., a true parent–offspring pair is not identified). A trade‐off exists between these two forms of error and the selection of an appropriate threshold for the LLR, *c*, is an important step for determining an acceptable rate for each form of error. An overly restrictive (i.e., large) *c* will minimize the false positive rate at the cost of increasing the false negative rate and a value of *c* that is too lenient (i.e., small) will minimize the false negative rate while increasing the false positive rate. The value of *c* should be determined on a case‐by‐case basis for datasets because it will be dependent upon project‐specific factors including number and variability of markers, the number of sampled adults, the number of sampled offspring, and the user's willingness to accept each type of error.

A final consideration that influences the estimate of false positive rates is the relatedness of the potential parents. Intuitively, if the sampled parents are highly related then there is greater chance of misassignment than when the potential parents are unrelated. The false positive error rate, *α*, is dependent upon the true relationship between the pair of individuals being assessed (Anderson & Garza, 2006). As such, we estimate error rates separately for an individual offspring and individuals of four different levels of relatedness: (a) unrelated individual, (b) aunt/uncle (sibling of the true parent), (c) half‐aunt/uncle (half‐sibling of true parent), and (d) first cousin of the true parent.

A simple Monte Carlo estimator of the false positive rate, *α*, would be to simulate pairs of genotypes given the true relationship, and then record the number of offspring which incorrectly assign given *m* and *c*. False positive rates are frequently small, but even small rates can be meaningful. A small per‐comparison false positive rate can accumulate into a large experiment‐wide value when a project evaluates large numbers of parents or offspring, because the number of potential parent–offspring pairs considered in an analysis is the product of the number of sampled parents and the number of sampled offspring. The false positive rate, particularly for unrelated pairs, is typically small enough that this simple estimator is computationally infeasible. Previous approaches to this issue have used importance sampling (Baetscher et al., 2018), but we implemented an alternative approach of stratified sampling to reduce Monte Carlo variance similar to the approach utilized by Delomas and Campbell (2021) for grandparent–grandchild trios. The domain of the estimator was stratified by the number of observed MIs in a potential parent–offspring pair. Because only pairs with the number of MIs equal to or below *m* are considered, the false positive rate in strata greater than *m* is zero and so they do not need to be sampled. The estimate of *α* is calculated as

$$\alpha \approx \sum_{j=0}^{m}\frac{p_{j}}{n_{j}}\sum_{k=1}^{n_{j}}I\left\{r_{\mathrm{jk}}\geq c\right\},$$

where $p_{j}$ is the size of each stratum, $n_{j}$ is the number of pairs simulated in stratum *j*, $r_{\mathrm{jk}}$ is the LLR for simulated pair *k* in stratum *j*, and *I* is an indicator function returning one when the condition is true.

The size of a stratum ($p_{j}$) is the probability that a pair of samples with the given true relationship (e.g., unrelated pair, aunt and niece, etc.) for which *α* is being calculated has the corresponding number of observed MIs. Anderson (2012) described a Markov chain modelling the observed number of MIs given genotype frequencies (incorporating missing data as a unique genotype) and genotyping error rates. This model is modified here to account for the common practice of only analyzing samples given a maximum number of missing genotypes. The maximum number of missing genotypes allowed in the current analyses was 10% of the loci. Let $s_{i}$be the state, describing the number of observed MIs and missing genotypes, after locus *i* in the Markov Chain. Prior to observing any loci, $s_{0}=\left(0,0,0\right)$, representing the number of observed MIs and number of missing genotypes for the two individuals, respectively. Let $a_{i}$ be a vector indicating whether an MI or missing genotypes are observed at locus *i*, in the same order as $s_{i}$. Because MIs can only be observed when neither genotype in the pair is missing, $a_{i}\in \left\{\left(0,0,0\right),\left(1,0,0\right),\left(0,1,0\right),\left(0,0,1\right),\left(0,1,1\right)\right\}$. Assuming the probability of a genotype being missing at a given locus (i.e., locus‐specific rate of missing genotypes) is known and that observation of missing genotypes is independent across loci and individuals, the probabilities of each possibility for $a_{i}$ can be calculated according to standard probability arguments. The probability of being in state x after a given locus can then be calculated as

$$P\left(s_{i+1}=x\right)=P\left(s_{i}\right)\sum_{a}P\left(a_{i+1}\right)I\left\{s_{i}+a_{i+1}=x\right\}.$$

This can be evaluated recursively to obtain the probabilities of each final state. The size of each stratum can then be calculated given a maximum number of missing genotypes. However, computer memory constraints can make saving all the probabilities impractical even with moderate numbers of loci. The probability of observing an individual with more than the allowed number of missing genotypes can be obtained through a similar process (but s and a now only represent missing genotypes in one individual). Because we assume that missing genotypes are independent between individuals, the probability of both samples having a valid number of missing genotypes is then straightforward to calculate and only the probabilities of states with *m* or fewer MIs and with both individuals having an allowable number of missing genotypes need to be saved.

In order to utilize the Monte Carlo estimator of *α*, genotypes for pairs must be simulated in relevant strata. The backwards algorithm can be used with this model to simulate genotypes. Given *L* loci, a value of $s_{L}$ is chosen within a stratum by sampling a categorical distribution with

$$P\left(s_{L}|\text{stratum}\right)\propto P\left(s_{L}\right),$$

for all $s_{L}$ in the stratum. Next, for each locus, iterating backwards, a value for $a_{i}$ is chosen by sampling a categorical distribution with

$$P\left(a_{i}|s_{i+1}\right)\propto P\left(a_{i}\right)P\left(s_{i}=s_{i+1}‐a_{i}\right).$$

Once $a_{i}$ is chosen, genotypes are sampled using the genotype frequencies for the pair calculated from the allele frequencies, Hardy–Weinberg equilibrium, and laws of Mendelian inheritance, and the rates of genotyping error. If both genotypes are observed ($a_{i}\in \left\{\left(0,0,0\right),\left(1,0,0\right)\right\}$), then the pair of genotypes are either sampled conditional upon an MI being present or not.

### 2.6 Experiment‐wide error rate estimation

**FIGURE 1:**
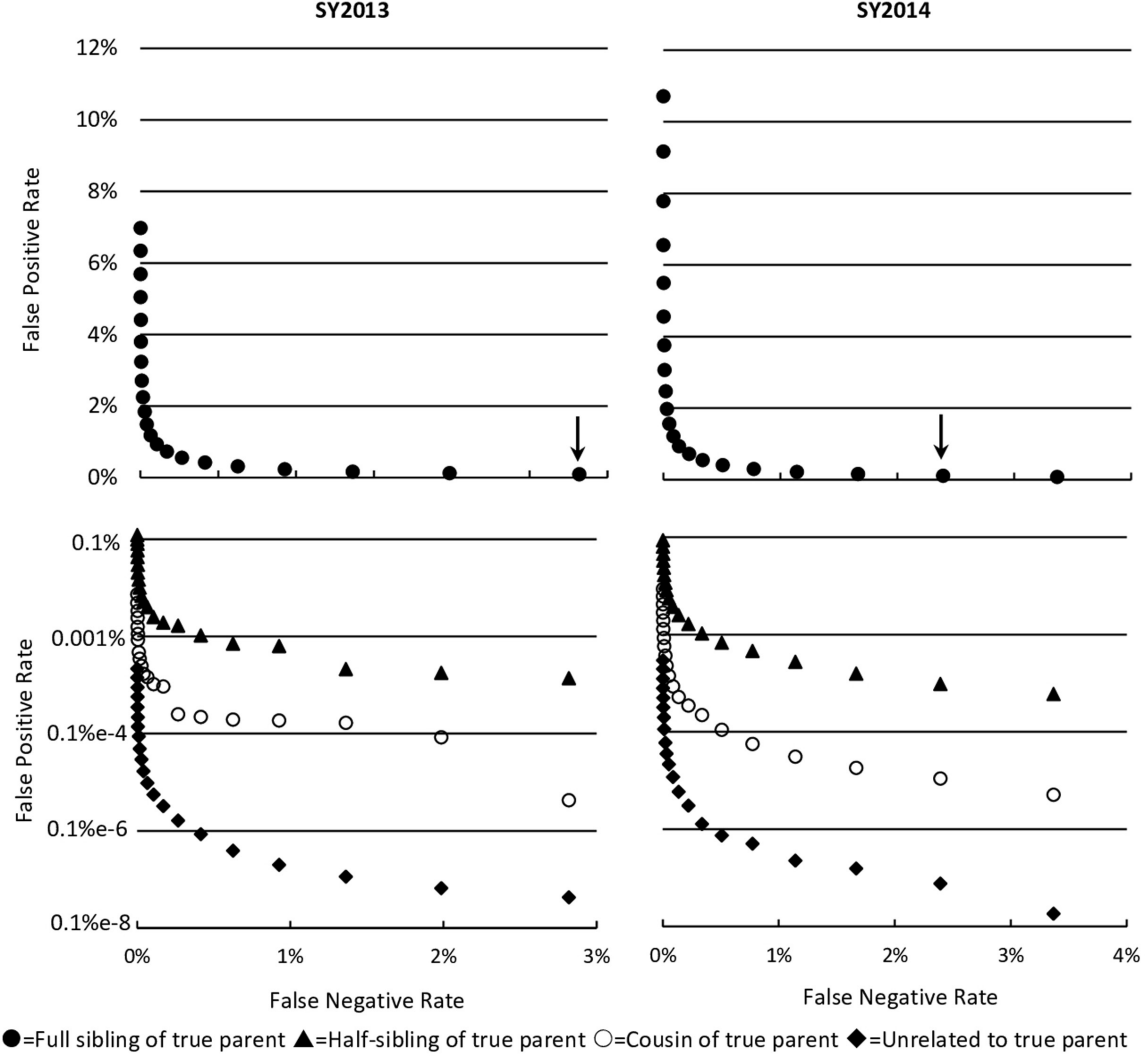
Simulated single‐parentage error rates for the SY2013 and SY2014 parental collections. Error rates for each of four levels of relatedness with the true parent are presented. Note difference in scale of *y* axes. Each point represents a corresponding LLR value, ranging from 0 to 20, associated with potential single parentage assignments. Arrows indicate assignment thresholds selected (LLR of 20 for SY2013 and 19 for SY2014) that allow for an acceptable per‐comparison false positive rate and overall false negative rates for each parental dataset

To fully estimate experiment‐wide false positive rates within a dataset requires the calculation of the per‐comparison error rate under each level of relatedness as described above. It also requires estimating the probability that a potential parent in a comparison is the full‐sib, half‐sib, or cousin of the true parent. The most comprehensive estimate of experiment‐wide error rates can be estimated as

$$N\left(P\left(\text{unrelated}\right)E_{u}+P\left(\text{aunt}\right)E_{a}+P\left(\text{half aunt}\right)E_{\text{ha}}+P\left(\text{cousin of parent}\right)E_{\text{pc}}\right)$$

where N is the total number of comparisons, $P\left(\mathrm{unrelated}\right)$ is the probability that a given comparison contains unrelated individuals, $E_{u}$ is the false positive rate for unrelated individuals, $P\left(\mathrm{aunt}\right)$ is the probability that a given comparison contains an aunt/uncle and niece/nephew, $E_{a}$ is the false positive rate for aunt/uncle and niece/ nephew comparisons, and similarly for half‐aunt and cousin of true parent. This assumes that no relationships other than the four described are present at meaningful levels. However, as we show later, the only category of relatedness that is meaningful for our study is $E_{a}$, the aunt/uncle error rate (i.e., when the full‐sibling of the true parent is evaluated). All other error rates are too small to be of concern (Figure 1). Because this sockeye population is highly managed and recovery efforts include applying genetic‐based parentage assignments to all released individuals (Kozfkay et al., 2019), we were able to simply enumerate the number of full‐siblings in each spawn year based on their parentage assignments. We then used this information to estimate the probability ($P_{\mathrm{fs}}$) that a single‐parentage comparison in the assignment analysis would contain the full‐sibling of a true parent and its offspring (i.e., aunt/uncle – niece/nephew) using the formula

$$P_{\mathrm{fs}}=\frac{2\left(\text{mean fullsibling family size}‐1\right)}{\text{number of sampled parents}}.$$

This is the expected number of aunts/uncles divided by the total number of parents, assuming all potential offspring are offspring of the potential parents and that all matings were between unrelated individuals. If some potential offspring were produced by an unsampled population (e.g., the resident population), then this estimate is biased high and leads to an overestimate of the error rate. We then incorporated this probability into an estimate of the experiment‐wide false positive error rate ($F_{p}$) for each application of the single‐parentage methodology:

$$F_{p}=N_{p}*N_{o}*\alpha_{\text{LLR}}*P_{\mathrm{fs}}$$

where $N_{p}$ is the number of sampled parents, $N_{o}$ is the number of sampled offspring, and $\alpha_{\mathrm{LLR}}$ is the false positive rate for the selected value of *c* (LLR assignment threshold).

### 2.7 False negative error rate estimation

We do not expect false negative rates to drive the selection of an LLR threshold for analyses and generally expect false negative rates to be low in most cases (5–10%, or even lower) given the power of modern genetic panels. As long as false‐negative rates are not excessively high we recommend that users minimize the false positive rate and simply take their false negative rate into account during interpretation. We adopted the approach of Anderson and Garza (2006) used for paired‐parent assignments and estimate false negative rates with a typical Monte Carlo routine. Observed genotypes were simulated for parent–offspring pairs and the proportion that were not assigned given *m* and *c* was recorded. Genotypes were sampled at each locus using probabilities for each genotype pair calculated using allele frequencies, the laws of Mendelian inheritance, and genotyping error rates. Genotypes were chosen to be missing in each individual (given a maximum number of missing genotypes per individual) using a similar forward–backward algorithm to the one described above but with the state only representing the number of missing genotypes in an individual.

## 3 RESULTS

### 3.1 Assignment patterns

Over 99.5% of sampled offspring genotyped successfully (Table 1). Three SNPs (One_1a.43386‐37, One_STC‐410, and One_UCA‐24) failed to genotype in all offspring samples and the average genotyping success rate for offspring across the remaining SNPs was 97.9%. Offspring were analyzed first with a paired‐parent analysis and any unassigned individuals were then subsequently analyzed with the single‐parentage analysis. Approximately one‐half of the successfully genotyped offspring received either a paired‐parent assignment (51.2%) or a single‐parent assignment (6.4%), but a large proportion of offspring (42.3%) received no kind of parentage assignment (Table 1).

All adults released in 2013 (male‐biased sex ratio) were successfully genotyped ensuring that any offspring produced by released parents could be identified as such and that all single‐parentage results can be interpreted as a mating between a released fish and a resident fish. One SNP (One_1a.43386‐37) failed to genotype in all parental samples and the average genotyping success rate for parents across the remaining SNPs was 97.3%. Parentage analyses detected 27 (25.0%) females from the 108 released that successfully reproduced. Of these, 22 (81.4%) were part of a paired‐parent assignment, three (11.1%) were part of a single‐parent assignment, and two (7.4%) occurred in both single‐ and paired‐parent assignments. Of the 238 released males, parentage analyses detected 51 (21.4%) that successfully reproduced. Of these, 21 (41.2%) were part of a paired‐parent assignment, 28 (54.9%) were part of a single‐parent assignment, and two (3.9%) occurred in both assignments (Table 2).

**TABLE 2:** Reproductive patterns of sockeye salmon adults released into Redfish Lake, Idaho for volitional spawning under a male‐biased sex ratio (2013) and a nearly‐equal sex ratio (2014)

Release year	Sex	Num. released	Num. (Prop.) successful	Inferred reproductive pattern		
				Assort. mating (paired parent.)	Disassort. mating (single parent.)	Both strategies
2013	Male	238	51 (21.4%)	21 (41.2%)	28 (54.9%)	2 (3.9%)
	Female	108	27 (25.0%)	22 (81.4%)	3 (11.1%)	2 (7.4%)
2014	Male	977	306 (31.3%)	303 (99.0%)	0 (0.0%)	3 (1.0%)
	Female	1094	387 (35.4%)	304 (78.6%)	22 (5.7%)	61 (15.8%)

Note

Reproductively successful parents were identified through parentage analyses of emigrating smolts. Reproductive patterns of released individuals were inferred based on the kind of parentage analysis that resulted in their detection. Parents detected in a paired‐parent assignment indicate assortative mating between released individuals. Parents detected only through single‐parentage analyses indicate disassortative mating between a released adult and an unsampled resident fish. Parents detected in both analyses indicate both reproductive strategies. Patterns indicate a reproductive shift by males under the two scenarios. Males employed an assortative mating strategy almost exclusively under an equal sex ratio but under a male‐biased sex ratio the majority shifted to a disassortative mating strategy in which they tended to spawn with smaller unsampled resident females.

Of the adults released in 2014 (equal sex ratio), 13 failed to genotype successfully (seven females and six males), thus 2058 (99.4%) of the parents were used in the parentage analysis for this spawn year. This results in an estimate of 98.8% of offspring (calculated as the proportion successfully genotyped males multiplied by the proportion successfully genotyped females) produced by the released fish being detectable in a paired‐parent assignment. The remaining proportion of offspring (1.2%) would not be detectable in a paired‐parent analysis and might instead receive a single‐parent assignment or fail to receive any kind of parentage assignment. One SNP (One_1a.43386‐37) failed to genotype in all parental samples and the average genotyping success rate for parents across the remaining SNPs was 96.3%. Of the 1094 released females, parentage analyses detected 387 (35.4%) that successfully reproduced. Of these, 304 (78.6%) were part of a paired‐parent assignment, 22 (5.7%) were part of a single‐parent assignment, and 61 (15.8%) occurred in both single‐ and paired‐parent assignments. Of the 977 released males, parentage analyses detected 306 (31.2%) that successfully reproduced. Of these, 303 (99.0%) were part of a paired‐parent assignment, no males were detected exclusively from a single‐parent assignment, but three males (1.0%) occurred in paired‐ and single‐parent assignments (Table 2).

The reproductive pattern of the released males shifted markedly under the male‐biased sex ratio with fewer males displaying an assortative mating pattern with the released females (*z* = −13.2; *p* < .01). Males identified through the single‐parentage analysis (disassortative spawners) were significantly (*t* = 7.6, *p* < .001) smaller (range = 323–534 mm FL, mean = 400.8 mm FL ± 47.6 mm *SD*, *N* = 28) than assortative males identified through the paired‐parentage assignment (range = 394–627 mm FL, mean = 521.8 mm FL ± 62.3 mm *SD*, *N* = 21). Disassortative males were also significantly (*t* = 5.8, *p* < .001) younger (range = 3–4 years, mean = 3.07 years ± 0.26 years *SD*, *N* = 28) than assortative males (range = 3–5 years, mean = 3.9 years ± 0.7 years *SD*, *N* = 21). Most (93%) of disassortative males were of the youngest age category (3 years old) while just 29% of the assortative males were 3 years old. None of the disassortative males were of the oldest age class (5 years old) while 19% of the assortative males were of this age group. Disassortative males were also smaller at a given age than the assortative males. Disassortative 3‐year‐old males were on average 48 mm smaller than 3‐year‐old assortative males and disassortative 4‐year‐old males were on average 25 mm smaller than 4‐year‐old assortative males (Table 3). No males in 2014 used a disassortative mating pattern exclusively.

**TABLE 3:** Age and fork length (FL) of male sockeye salmon released in 2013 under a male‐biased sex ratio and in 2014 under an equal sex ratio

Year	Mating pattern	Age	Number	Avg. FL (mm)	Overall Avg. age (years)	Overall Avg. FL (mm)
2013	Disassortative	3	25	391.4	3.07	400.8
		4	2	518.8		
	Assortative	3	6	439.3	3.90	521.8
		4	11	543.7		
		5	4	585.0		
2014	Assortative	3	103	440.7	3.67	504.0
		4	178	538.7		
		5	7	554.6		

Note

Males that mated with released females are categorized as “Assortative” and while males that mated with the resident females are categorized as “Disassortative.” Disassortative males were younger and smaller than assortative males. Length data was missing for one 3‐year‐old disassortative male in 2013 and is excluded from the table. No males in 2014 displayed a disassortative pattern exclusively, therefore only assortative males are summarized. Age could not be determined for 15 of these males and they are excluded from the table.

### 3.2 Evaluating single‐parentage error rates

The SY2013 parental dataset contained 337 variable SNPs, and the SY2014 dataset contained 347 variable SNPs. In each dataset the false positive error rate declined quickly for the four levels of relatedness and approached zero as higher LLR thresholds were evaluated (Figure 1). In three levels of relatedness (half‐sibling, cousin of parent, and unrelated) the rate of false positives quickly becomes negligible and only the error rate for full‐siblings remained a concern at some levels of LLR. Our main priority was to select an LLR threshold that minimized the false positive rate while allowing for an acceptable false negative rate. We selected a minimum LLR threshold of 20 for the SY2013 dataset and a threshold of 19 for the SY2014 dataset (Figure 1). No fish were observed in these analyses that had multiple single‐parentage assignments with an LLR greater than the chosen values for *c*. The estimated error rates associated with the LLR are a per‐comparison rate; therefore, the estimate of the experiment‐wide error rate (i.e., the total number of false positives expected in these analyses) can be calculated by multiplying together the error rate, the probability of an evaluation containing the full‐sibling of the true parent ($P_{\mathrm{fs}}$), the number of parents sampled, and the number of offspring evaluated. For example, our LLR threshold of 20 is estimated to produce 5.3 false positives for the SY2013 dataset and the LLR threshold of 19 is estimated to produce 6.4 false positives for the SY2014 dataset (Table 4). These false positives will have a diminishing effect on this study because we evaluate whether a parent was reproductively successful and not the magnitude of that success. In other words, if a parent is identified correctly via a single‐parentage analysis then any additional false positive assignments to the same parent do not affect the interpretation of the reproductive patterns. It is also worth noting the expected number of false positives in our analysis is low despite a high proportion of related individuals present in the parental population of released fish (Table 5).

**TABLE 4:** Per‐comparison false positive rates and the estimated number of analysis‐wide false positive assignments for each dataset

Dataset	False Pos. rate	Num. parents	Num. offspring	$P_{\mathrm{fs}}$	Total Est. false Pos.	Total S‐P assignments
SY2013 Sockeye	0.00087	346	1852	0.0095	5.3	94
SY2014 Sockeye	0.00115	2071	1040	0.0026	6.4	219

Note

False positive rates are derived from Figure 1. The number of offspring evaluated are the individuals that did not receive a paired‐parent assignment and were evaluated with the single‐parent assignment methodology. $P_{\mathrm{fs}}$ is the probability that a parent‐offspring evaluation contains a full‐sibling of the true parent and is estimated using the family sizes of full‐siblings present in the parental dataset (see text). The total number of estimated false positive assignments is equal to the product of the four preceding numbers in the table. The total number of single‐parentage assignments accepted for each dataset is also presented.

**TABLE 5:** Distribution of various categories of relatedness among the parental fish released in 2013 and 2014

Relationship	Release year	
	2013	2014
Full siblings	98 (28%)	1705 (82%)
Half siblings	145 (42%)	153 (7%)
Unrelated	75 (22%)	115 (6%)
No info	28 (8%)	98 (5%)
Total	346	2071

Notes

Relationships were determined from routine parentage assignments conducted annually on maturing adults as part of ongoing recovery efforts (see Kozfkay et al., 2019). Despite large proportions of highly related individuals in each parental dataset the single‐parentage analysis is expected to generate a low number of assignment errors (Table 4).

## 4 DISCUSSION

To test for disassortative mating by male sockeye salmon released into Redfish Lake we used a combination of paired‐parent and single‐parent assignments to calculate and compare the proportion of successful male spawners under scenarios of equal and male‐biased sex ratios for the released adults. Parentage assignments from paired‐parent analyses were used to identify parents that mated assortatively and spawned with other released fish, while parentage assignments from single‐parentage analyses identified parents that spawned disassortatively with unsampled resident fish. The parentage assignments demonstrate that male sockeye salmon changed their reproductive behavior in response to the sex ratio of released fish. This pattern is consistent with several predictions in behavioral ecology of salmonids. First, deviations from an equal sex ratio often result in different reproductive behaviors than under an equal sex ratio **(**Emlen & Oring, 1977; Weir et al., 2011 **)**. Second, disassortative mating patterns by some males supports predictions that intrasexual competition under a male‐biased sex ratio excludes those individuals from spawning with the females released into the lake (Foote, 1988; Hanson & Smith, 1967). Finally, the phenotypes of the disassortative males are consistent with previous observations in this species (Fleming & Gross, 1994; Quinn & Foote, 1994), in which the largest males tend to spawn with the largest females, relegating the smaller males to spawn with the population of resident females.

When the sex ratio of the released fish was equal then all reproductively successful males spawned with the released females (Table 2). This reproductive pattern was mirrored by the reproductively successful released females, of which 94.3% spawned with a released male (i.e., identified through paired‐parentage analysis). None of the males were detected exclusively through the single‐parent analysis, indicating limited spawning with the population of unsampled resident females. Under an equal sex ratio there was reduced intrasexual competition among males. In fact, the slight bias in the number of females in this scenario likely ensured that most males had an opportunity to spawn with one of the released females. These patterns indicate strong assortative mating by both sexes among the released fish and are consistent with previous observations of size‐assortative mating within sockeye salmon (Foote, 1988; Hanson & Smith, 1967).

Fewer males displayed an assortative mating pattern under a male‐biased sex ratio. Instead, most reproductively successful males (54.9%) in this scenario were identified only through the single‐parentage analysis, indicating a tendency to mate disassortatively with unsampled resident females. In contrast, most of the released females that were reproductively successful (81.4%) were identified through paired‐parent analysis, indicating that they exhibited assortative mating and spawned with released males. These patterns occurred despite similar proportions of reproductively successful males and females (Table 2). Under the male‐biased sex ratio the number of successful spawnings for males appears to be limited by the number of released females. Dominant male *O*. *nerka* are known to guard their mates (Morbey, 2002) which likely forced many males to seek spawning opportunities with the population of smaller resident females. Female salmonids often prefer to mate with larger males (Berejikian et al., 2000; Foote, 1989; Labonne et al., 2009; Maekawa et al., 1994; Neff et al., 2008). Males that were smaller and younger than the dominant males were still approximately twice the size of the resident males which likely facilitated their successful spawning with smaller resident females (Table 3).

The reproductive patterns observed for released females raise questions about the evolutionary role of the resident life history for this population. The situation involving life history forms in Redfish Lake has been described as “unusually complex for the species” (Waples et al., 2011) and little is known about this resident population. The resident males may represent a disruptive strategy seen in other salmonids (Berejikian et al., 2010; Gross, 1985) in which smaller males have increased fitness in years of abundant anadromous returns and experience a significant degree of success for this trait to persist (Smallegange & Johansson, 2014). In each spawn year some of the released females were identified in both paired‐ and single‐parent analyses indicating that they spawned with a released male but also with a resident male that presumably employed a sneaker or satellite strategy (Table 2). The proportion of females that were exposed to a sneaker strategy by resident males varied among years but appears to be correlated with the abundance of the released females and also with the magnitude of intrasexual competition by males. The large number of females (*n* = 1094) released into the lake in 2014 resulted in 15.8% of females being detected in both single‐ and paired‐parent assignments. Alternatively, the small number of females (*n* = 107) released into the lake in 2013 resulted in only a small fraction of females (3.8%) being detected in both types of parentage analyses (Table 2). This suggests that resident males may fertilize a larger proportion of the full‐sized female's eggs as these females become more abundant on the spawning ground. However, the reproductive pattern of females could also be explained by varying degrees of competition among males. In 2013, the presence of multiple dominant males per female appeared to be more effective at excluding sneaker males than under conditions where males and females were more equally represented on the spawning ground in 2014. Understanding these interactions between resident males, full‐sized females, and the sex ratio of released fish will be important in guiding recovery strategies of this population.

These results are encouraging for the recovery efforts of this endangered population because it indicates that an abundance of released males can still contribute to the production of anadromous offspring by spawning with the resident population. Exclusion of some males from the spawning population is expected, but without a resident population a male‐biased sex ratio in the released fish would likely have resulted in even more of the released males from contributing reproductively. Releasing an equal sex ratio appears to result in a higher proportion of each sex being reproductively successful (Table 2). However, the presence of the resident population provides additional reproductive opportunities for fish that might otherwise be excluded from spawning. Additionally, this disassortative mating helps to maintain genetic connections between the different life histories that contribute to the production of anadromous smolts.

The findings of this study were possible because of the application of the single‐parentage assignment methodology. Our method has the advantage of helping researchers to estimate project‐specific assignment errors which we show can be controlled through selection of an appropriate assignment threshold (*c*). The single‐parentage methodology, however, is not without limitations and quantifying error rates is an important step in the process. This study system was particularly well positioned for evaluating the experiment‐wide false positive error rate ($F_{p}$) because the population is well pedigreed which allows for direct measurement of the number of full‐sibling families within each parental dataset ($P_{\mathrm{fs}}$). Other researchers that apply our methodology may have to estimate the relatedness among the potential parents using a sibship analysis with programs such as colony (Jones & Wang, 2010) as part of the process for estimating ($F_{p}$). Researchers might find that implementation of single‐parentage may be limited to study systems where the number of samples collected from the parental population or offspring is limited. The ability to estimate and control error rates should give researchers the confidence to apply this technique if these rates are found to be acceptably low.

Advances in the field of molecular ecology are driven by the development of new molecular markers as well as improvements in the analytical approaches for those markers. There is widespread interest in the study of reproductive behavior in a variety of other organisms including salmonids (Auld et al., 2019), mammals (Clutton‐Brock & McAuliffe, 2009), and birds (Wang et al., 2019) and molecular techniques for parentage analysis provide an unprecedented ability to study reproductive patterns in their natural populations. We show how single‐parentage analysis furthered our understanding of the reproductive behavior of this endangered salmonid and believe that this methodology has the potential to provide insights into the life histories of numerous other organisms.

## AUTHOR CONTRIBUTION

**Craig A. Steele:**Conceptualization (lead); Formal analysis (lead); Investigation (lead); Writing – original draft (lead); Writing – review & editing (lead). **Thomas A. Delomas:** Methodology (lead); Software (lead); Writing – review & editing (supporting). **Matthew R. Campbell:** Writing – review & editing (supporting). **John H. Powell:** Formal analysis (supporting); Supervision (lead); Writing – review & editing (supporting).

## Data Availability

The data that support the findings of this study are openly available in Dryad at: https://doi.org/10.5061/dryad.905qfttm1.
